# The Youth Mental Health Risk and Resilience Study (YouR-Study)

**DOI:** 10.1186/s12888-017-1206-5

**Published:** 2017-01-26

**Authors:** Peter J. Uhlhaas, Ruchika Gajwani, Joachim Gross, Andrew I. Gumley, Stephen M. Lawrie, Matthias Schwannauer

**Affiliations:** 1 Institute for Neuroscience and Psychology, University of Glasgow, 58 Hillhead Street, Glasgow, Scotland; 2 Mental Health and Wellbeing, Institute of Health and Wellbeing, University of Glasgow, Glasgow, Scotland; 3 Department of Psychiatry, University of Edinburgh, Edinburgh, Scotland; 4 Department of Clinical Psychology, University Edinburgh, Edinburgh, Scotland

**Keywords:** Adolescence, Psychosis, Early intervention, Biomarker, Affect regulation, Attachment

## Abstract

**Background:**

The transition from adolescence to adulthood is associated with the emergence of psychosis and other mental health problems, highlighting the importance of this developmental period for the understanding of developing psychopathology and individual differences in risk and resilience. The Youth Mental Health Risk and Resilience Study (YouR-Study) aims to identify neurobiological mechanisms and predictors of psychosis-risk with a state-of-the-art neuroimaging approach (Magnetoencephalography, Magnetic Resonance Spectroscopy, Magnetic Resonance Imaging) in combination with core psychological processes, such as affect regulation and attachment, that have been implicated in the development and maintenance of severe mental health problems.

**Methods/Design:**

One hundred participants meeting clinical high-risk criteria (CHR) for psychosis through the Comprehensive Assessment of At-Risk Mental State and Schizophrenia Proneness Instrument, Adult Version, in the age range from 16 to 35 years of age will be recruited. Mental-state monitoring up to a total of 2 years will be implemented to detect transition to psychosis. In addition, a sample of *n* = 40 help-seeking participants will be recruited who do not meet CHR-criteria, a group of *n* = 50 healthy control participants and a sample of *n* = 25 patients with first-episode psychosis. MEG-activity will be obtained during auditory and visual tasks to examine neural oscillations and event-related fields. In addition, we will obtain estimates of GABA and Glutamate levels through Magnetic Resonance Spectroscopy (MRS) to examine relationships between neural synchrony and excitatory-inhibition (E/I) balance parameters. Neuroimaging will be complemented by detailed neuropsychological assessments as well as psychological measures investigating the impact of childhood abuse, attachment experiences and affect regulation.

**Discussion:**

The YouR-study could potentially provide important insights into the neurobiological mechanisms that confer risk for psychosis as well as biomarkers for early diagnosis of severe mental health problems. Moreover, we expect novel data related to the contribution of affect regulation and attachment-processes in the development of mental health problems, leading to an integrative model of early stage psychosis and the factors underlying risk and resilience of emerging psychopathology.

## Background

The transition from adolescence to adulthood is associated with frequent mental health problems, ranging from affective disorders to more severe forms of psychopathology, such as psychosis [1]. Current data suggests that approximately one in every four to five youth will develop a mental disorder with severe impairment across their lifetime [2]. Accordingly, understanding the developmental trajectory as well as potential ways to identify early signs of serious mental health problems is a key priority of current research [3].

Among mental health problems with an onset during the transition from adolescence to adulthood, psychotic disorders, such as those labelled as schizophrenia (ScZ), are a particular challenge given that certain cognitive and physiological signals are already present prior to the manifestation of psychosis [4]. Because the actual onset of psychotic symptoms is frequently accompanied by an extended prodromal phase of up to 5–6 years [5], clinical high-risk criteria (CHR) have been developed that allow the identification of young people at risk of developing psychoses. Firstly, Ultra-high risk (UHR) criteria are based on the presence of attenuated, psychotic symptoms as assessed by the Comprehensive Assessment of At-Risk Mental State (CAARMS) instrument [6] or the Structured Interview for Prodromal Symptoms (SIPS) [7]. Moreover, UHR-criteria on the CAARMS and SIPS instruments include a genetic risk and deterioration syndrome as well as brief limited intermittent psychotic episodes (BLIPs).

Secondly, CHR have been developed based on the basic symptom (BS) concept proposed by Huber and colleagues [8]. From this perspective, self-experienced perceptual and cognitive anomalies represent the earliest manifestation of psychosis risk while psychotic symptoms are considered as an adaptive response to render the anomalous cognitive experiences coherent [8]. Recent data from several studies using the Schizophrenia Proneness Instrument, Adult Version (SPI-A), have shown that CHR-criteria based on BS alone can identify young people at-risk for the development of psychosis with transition rates of up to 54.9% within four years period [9]. Similar transition rates have been originally observed for UHR-criteria, although recent studies have indicated that transition rates may be decreasing, ranging between 10 and 30% [4]. Interestingly, the combination of both basic symptoms and UHR-criteria the predictive power [10].

While screening procedures are characterized by sufficient diagnostic accuracy to detect at-risk individuals [11], these approaches are currently not sensitive and specific enough to predict psychosis-risk on an individual level, a key objective for early intervention research. Accordingly, biomarkers may be required to boost prediction and furthermore to allow insights into the underlying neurobiology of the at-risk state. The search for underlying mechanisms und biomarkers is furthermore complicated by the fact that CHR-participants are a highly heterogeneous phenotype with genetic and environmental risk factors contributing to the at-risk state for psychosis, including trauma and affect dysregulation [12]. This is highlighted by recent findings that CHR-participants who do not convert to psychosis develop a range of psychiatric disorders, including affective and personality disorders [13]. Accordingly, the CHR-state may not only confer risk for the development of psychosis per se but more broadly for diverse psychopathology [14].

## Rationale

While data from neuroimaging and neurophysiology have demonstrated that many of the patterns observed in ScZ-patients are already present in at-risk individuals [4], the underlying neurobiological and psychological mechanisms that give rise to subthreshold psychotic symptoms and self-experienced cognitive and perceptual disturbances remain poorly understood.

The search for biomarkers for early diagnosis and prognosis in CHR-participants has been largely conducted with anatomical and functional Magnetic Resonance Imaging (MRI/fMRI), brain imaging techniques with excellent spatial resolution. While useful in delineating the architecture of functional and anatomical networks, these techniques only allow only indirect links to cellular and physiological mechanisms of circuit abnormalities [15]. Moreover, MRI approaches are unable to capture neural processes with high temporal resolution, which is essential for measuring fast rhythmic fluctuations of neuronal events that have been recently implicated in major psychiatric conditions [16].

Accordingly, the development of biomarkers may require a stronger focus on non-invasive techniques that allow a direct assessment of neuronal dynamics at high temporal resolution. This approach is supported by emerging evidence suggests that ScZ is associated with specific impairments in neural oscillations and their synchronization (neural synchrony), in particular at gamma-band frequencies (30–200 Hz) [15]. Brain oscillations have been shown to occur during normal brain functioning and are closely linked to the ability to perceive, memorize and attend to information [17–19]. Thus, it appears that brain oscillations could be a key to understanding the neurobiological mechanisms of psychosis-risk as well as provide biomarkers for predicting mental health outcomes, including psychosis, in CHR-individuals. Importantly, the impairments in neural synchrony are ideally suited for translational research because of evidence linking gamma-band oscillations during normal brain functioning to the integrity of GABAergic interneurons [20] and glutamatergic neurotransmission [21]. Supporting this hypothesis, the diagnosis of ScZ is associated with pronounced abnormalities in levels of GABA and Glutamate measured by magnetic resonance spectroscopy (MRS) [22]. Finally, blood and urine-samples will be collected to allow for potential genetic testing and proteomic analysis.

In addition to basic insights into the neurobiology of circuit dysfunction conferring risk for psychosis, the YouR-study will examine several core psychological processes involved in the maintenance and development of psychosis and several mental health problems, such as affectregulation and attachment. Evidence from behavioural and neuroimaging-studies suggests that problems in affect regulation may constitute a core risk factor in ScZ [23] which could be related to early trauma [24]. Preliminary evidence suggests that CHR-participants may experience greater difficulties in affect regulation [25] but the relationship to early trauma is unclear. Accordingly, we will further explore the role of affect regulation in at-risk participants and its relationship to psychosis as well as the role of attachment styles in the developmental trajectory of CHR-participants and its relationship to clinical variables.

## Aims

The primary aim of the YouR-study is to characterize changes in neural oscillations in CHR-participants and to develop a biomarker for psychosis-prediction and mental health outcomes. Accordingly, we will recruit a sample of *n* = 100 CHR-individuals for psychosis and apply MEG during cognitive tasks and resting-state activity in combination with advanced analytic tools to identify the frequencies as well as brain regions involved in aberrant neural synchrony. In addition, to establish links between neural synchrony and E/I-balance parameters MRS-measured GABA/Glutamate levels in auditory and visual cortices will be obtained. During a follow-up period of up to two years, transition to psychosis, functional status and changes in affect-regulation will be monitored. Moreover, the study will identify the relationship between disturbances in affect regulation, attachment and trauma and their contribution towards the development of psychosis.

## Methods/Design

The study will be a longitudinal cohort design with CHR-participants using neuroimaging to investigate brain activity and psychological measures to identify risk and resilience factors as well as biomarkers for emerging mental health problems. The YouR-Study will be performed according to the Research Governance Framework for Health and Community Care (Second edition, 2006).

## Recruitment and participants

We aim to recruit up to *n* = 100 participants meeting CHR-criteria in the age range from 16–35 years and we expect to identify from this participant group an additional *n* = 25 participants who meet criteria for FEP over a four-year period. The recruitment of the CHR- and FEP-groups will involve NHS-patients services in NHS Greater Glasgow and Clyde and NHS Lothian, NHS First Episode Psychosis Services, Community Mental Health Teams (CMHTs), Primary Care Mental Health Teams (PCMHTs), Clinical Psychology Services, Community Adolescent Mental Health Services (CAMHS), and Non-Governmental mental health organisations. Recruitment for the YouR-study began in October 2014 and is expected to continue until 12/2017.

In addition, the YouR-study recruits from the general population through a website http://www.your-study.org.uk which includes an initial screening through the 16-item version of the prodromal questionnaire (PQ) [26] and a 12-item scale of perceptual and cognitive items. Previous data suggested that a cut-off score of 6 or more positively answered items on the 16-item version of the PQ produced correct classification of CHR-criteria based on CAARMS-interviews with high sensitivity and specificity [26].

CHR- and FEP-groups will be compared to two groups of participants: 1) A group of *n* = 40 help-seeking participants below the threshold for UHR- or SPI-A criteria who will be recruited through the same recruitment pathways as participants meeting CHR-criteria (low-risk group). The reason for the inclusion of this group is to control for non-specific factors associated with CHR-individuals, such as the presence of co-morbid non-psychotic psychopathology, drug abuse and lower educational status. And 2) A group of *n* = 50 participants without the presence of any current DSM-IV disorder, current substance abuse and first-degree relative with psychosis.

## Clinical-assessments and CHR-criteria

To establish CHR-criteria, the CAARMS-Interview [15, 16] and the COGDIS/COPER items for the SPI-A [27] are administered. Participants are recruited into the CHR-group if they meet a) SPI-A COGDIS/COPER-criteria b) ARMS attenuated psychosis group (subthreshold psychotic syndrome present in the last year without a decline in functioning) c) ARMS vulnerability group (family history of psychosis plus a 30% drop in GAF) and d) ARMS BLIPs-group (brief limited intermittent psychotic symptoms). In addition, the M.I.N.I. International Neuropsychiatric Interview (M.I.N.I. 6.0) [28] will be administered to identify psychiatric comorbidity as well as the scales for premorbid adjustment [29] and social and functional role scale will be administered [30]. Previous studies indicated that these variables are significantly impaired in CHR-participants and are predictors for transition to psychosis and functional outcome [31–33].

## Neuropsychology

CHR-participants are characterized by cognitive deficits in several domains, including processing speed and verbal memory [34], which are predictors for transition to psychosis [35]. The neuropsychological assessment of the YouR-study consists of the Brief Assessment of Cognition in Schizophrenia Battery (BACS) which has been extensively used in ScZ-research [36]. In addition, the following tasks from the University of Pennsylvania Computerized Neuropsychological Testing Battery (PennCNP [37]: a) Continuous Performance Test b) N-Back Task and c) Emotion Identification Task will be included in the neuropsychological assessment. Finally, the Edinburgh Handedness Inventory [38] and the National Adult Reading Test [39] will be administered.

## Questionnaires

Several psychological measures will be used in order to identify mechanisms of change and predictors of transition to psychosis and outcome. All are brief self-report scales, which have good psychometric properties. These include: 1) The Beliefs About Paranoia Scale (BAPS) [40] 2) The Brief Core Schema Scale (BCSS) [41] 3) The Psychosis Attachment Measure (PAM-SR) [42] 4) Adverse childhood experience scale (ACES) [43] and 5) The Rust Inventory of Schizotypal Cognitions (RISC) [44] 6) Inventory of interpersonal problems [45]– 32 item Version 7) The Significant Others Scale [46] and 8) The International Positive and Negative Affect Schedule, short-form (I-PANAS-SF) [47] and 9) Social Interaction Anxiety Scale [48] and the Functional Remission in General Schizophrenia (FROGS) scale [49].

## Follow-up

Follow-up interviews will be conducted every 3–6 months with CHR and low-risk participants. This will include subscales of the CAARMS as well as questionnaires to examine stress-levels, interpersonal functioning and affect regulation. In addition, the SCID I interview and the social and functional role scales will be added at follow-up appointments at 6, 12 and 24 months.

## Outcome parameters

In addition to conversion to psychosis in CHR-individuals, the YouR-study will examine changes in global functioning as well as role and social functioning as important outcome parameters in CHR-participants.

## Neuroimaging

### MEG

All neuroimaging experiments will be conducted within the facilities of the Centre for Cognitive Neuroimaging (CCNi), University of Glasgow. MEG-data will be recorded with a whole-head 248-channel 4D Neuroimaging WH3600 system at 1017.25 Hz sampling-rate and filtered with an online 400 Hz low-pass filter (DC-400 Hz).

MEG signals will be pre-processed and analyzed using the open source matlab-toolbox fieldtrip [50] and customized scripts. Data-analyses will be conducted at sensor and source-level in the 1–150 Hz frequency range. Time-frequency representations will be estimated through Morlet-Wavelets and Multi-tapers. In addition, event-related fields (ERFs) during auditory mismatch negativity (MMNm) and sensory attenuation will be examined. MEG is ideally suited for the analysis of source-activity and source-reconstruction techniques, such as beamformers [51], and Minimum Norm Estimates (MNE) will be employed [52].

We will employ individual head models using a common dipole grid in MNI space. Cortical segmentation and surface reconstruction are performed using SPM8 as well as spatial alignment of structural MRIs. In a second step, we will select peak voxels for the reconstruction of virtual channels which will allow the examination of the time course of oscillatory activity within a specified frequency band and the analysis of functional connectivity with measures such as Partial Directed Coherence (PDC) [53], Granger Causality [54] and Phase-Lagged Synchronization [55].

### Experimental tasks

Resting-state measurement: We will obtain resting-state data in an eyes-open condition (duration: 4 min) which provides important evidence on the organization of large-scale networks and their impairments.

Visual Grating Task: We will employ a visual task that was designed to elicit robust high-frequency activity in the context of focused attention [56]. On each trial, participants are shown a circular sine wave grating at central screen fixation that contracts towards the fixation point. Participants are required to press the response button with their right index finger when the stimulus increases in velocity which occurs between 750 and 3000 ms post stimulus onset (10% of the trials are catch trials in which no acceleration occurred). Extensive evidence suggests that high-frequency oscillations are robustly elicited by this paradigm with a signal-increase at the source-level of ~200% and excellent test-retest reliability in controls measured with MEG-data [57]. Moreover, data in healthy controls [58] and invasive electrophysiological data [59] suggest tight correlations of gamma-band fluctuation with reaction times (RTs) RTs as well as with detection rates in ScZ-patients [60].

An auditory steady-state (ASS) paradigm: Kwon et al. [61] conducted the first 40 Hz ASSR-study in ScZ-patients using EEG and reported reduced power and synchronization to 40 Hz stimulation. These initial findings have been replicated by several groups in EEG- and MEG-recordings (for a review see [62]). Participants are presented auditory stimuli consisting of 2000-msec 1000 Hz carrier tones, amplitude modulated at 40 Hz (100 trials). In addition to the passive presentation of ASS-stimuli, participants will be asked to initiate the same auditory stimuli through button press which allows for a comparison between auditory responses during a self-initiated sensory processing vs. passive stimulation or (sensory attenuation) [63]. Evidence suggests that both ScZ-patients as well as CHR-individuals are impaired in sensory attenuation [64].

Auditory MMNm Paradigm: MMN is an event-related potential/field elicited automatically by violations of previously established auditory regularities [65]. MMN amplitudes have been consistently found to be attenuated in both medicated and unmedicated ScZ- patients [66] Moreover, recent studies have found MMN signals to be present already in CHR-participants, indicating that MMN-deficits could represent a biomarker for psychosis development [67].

The YouR MEG-battery will investigate MMNm amplitude responses to both duration deviants and sound omissions during an auditory oddball paradigm in which three different sequences of auditory stimuli will be presented binaurally at ~ 70 dB (150 ms SOA, 700–1000 ms ISI). In addition, an omission sequence which contains only four identical tones is included to examine auditory predictions. Participants are instructed to focus their attention away from the sounds and to perform a simple visual detection task.

### MRS-spectroscopy

We will examine both GABA and Glutamate/Glutamine (Glx) levels with 1H-MRS in three 2 × 2 × 2 cm^3^ single-volume ROIs 1) right visual cortex and 2) left and right auditory cortex to examine correlations between fluctuations in neural oscillations GABA/Glutamate levels. 1H-MRS spectra will be acquired on a 3 T Siemens Magnetom Tim Trio MRI Scanner, using a 32-channel radiofrequency head coil and a MEGA point-resolved spectroscopy (PRESS) spectral *J-difference* editing sequence. The following parameters will be used: TR/TE 1500/68 ms, 1200 Hz acquisition bandwidth, vector size of 512, 256 averages (128 editing-on and 128 editing-off scan) and a delta frequency of −1.7 (on scan; special symmetrical editing approach to remove contamination of co-edited macromolecules at 1.9 ppm) and 7.5 ppm (off scan).

To assess glutamate levels, both physiologically active and inactive glutamate are measured (Glu). The proposed sequence (MEGA-PRESS) allows the detection of an aggregated signal representing the sum of glutamate and glutamine (Glx). Because the majority of physiologically active glutamate is derived from glutamine, high levels of Glx suggest elevated glutamatergic activity. We expect reduced GABA-levels but elevated Glx levels in the CHR-group which correlate with reduced power and peak-frequency of γ-band activity.

### Anatomical imaging: MRI/DTI

T1 weighted anatomical data will be acquired using a 3D MPRAGE sequence (FoV = 256x256x176 mm3, voxel size = 1x1x1 mm3, TR = 2250 ms, TE = 2.6 ms, TI = 900 ms, FA = 9°). For the analysis of MRI, we will focus on measures of cortical thickness as well as volume of gray and white matter. In addition to whole brain analyses, we will focus on ROIs in the auditory and visual cortex to correlate alterations in structural variables with MEG-parameters.

Moreover, the YouR-study will acquire DTI-data with the following sequence: 2D slice selective spin echo EPI sequence with diffusion encoding, 89 axial slices with a thickness of 1.7 mm, TR/TE = 1200 ms/100 ms, bandwidth = 1346 Hz/pixel, echo spacing = 0.85 ms. For DTI analysis, 67 diffusion encoding directions with a b-value of 1000 s/mm2 will be covered.

## Sample size and power calculation

Our current research with MEG has demonstrated large effect sizes for deficits in high-frequency oscillations in chronically medicated ScZ-patients as well as in medication-naïve FE-ScZ patients (chronic ScZ: d = 1.26; FE-ScZ d = 1.0) [68, 69]. Because we will employ novel and more advanced analyses approaches for the proposed project, we are confident that we will maximise the possibility to detect dysfunctions in CHR-participants that will be in the range and above of effect sizes currently available for prodromal ScZ-research.

Previous studies with a variety of methods, such as MRI, fMRI as well as event-related potentials (ERPs), have demonstrated anatomical and physiological impairments in CHR-cohorts with medium to large effect sizes compared [70]. Electrophysiological parameters were among those deficits with the largest effects [67]. For example, Atkinson et al. [71] demonstrated an impairment in MMN in CHR-participants vs. controls of d = .75. Accordingly, given a sample of *n* = 50 controls and 100 CHR-participants and an estimated effect size of .75 for the current study, the power to detect significant differences in MEG-parameters between controls and CHR-participants is 97%.

In regards to the ability to distinguish between CHR-participants who will transition to psychosis vs. CHR-participants without conversions, previous published effect sizes have reported medium but also large effect sizes for differences on anatomical and functional parameters [70]. For the current study, a conservative, medium effect-size of d = .50 for a sample of *n* = 30 converted CHR-participants vs. 70 CHR-non converted CHR-participants will yield a statistical power to detect significant differences between converted vs. non-converted CHR-participants of 82%. The sample of *n* = 30 converted CHR-participants is consistent with a meta-analysis on conversion rates in CHR-participants over a two year period [4]. Should CHR-participants be lost in the follow-up period, we will recruit additional participants during the course of the project.

## Statistical analysis

Interim analysis of neuroimaging and clinical data will be carried out after the recruitment of *n* = 50 CHR, 25 low-risk and 25 control participants. We will systematically explore relationships between MEG-variables (task and resting- state) and GABA/Glutamate levels with psychopathological variables (CAARMS, SPI-A) and neuropsychology (BACS) in the CHR-group. Specifically, we will identify those MEG (sensor, frequency and source-regions) and MRS-parameters with the largest effect size and perform information theoretical analysis to identify linear and non-linear dependencies.

In addition to parametric analysis of group differences in baseline-variables, we will employ a multivariate machine learning technique towards the development of predictors for conversions and functional outcome in CHR-individuals, an approach which has been used recently in the neuroanatomical classification of ScZ [72]. In the first step, we will reduce the dimensionality of the MEG-data by focusing on reconstructed source-time courses with significant task effects (baseline vs. task). For resting-state data, we will employ a principal component analysis (PCA) to the source-reconstructed activity in the 4–200 Hz frequency range in order to reduce the number of variables.

To examine transitions, we employ a Generalized Linear Model (GLM) to assign MEG-data of individual subjects to one of the three designated categories (controls vs. transitioned CHR vs. non-transitioned CHR). Following a 10-fold cross validation, we will divide the total sample into 10 non-overlapping samples. Each sample will be iteratively held back as test data while the classifier is trained on the 9 remaining samples. A multiclass logistic regression (GLM) ranks features by their estimated effect on the resulting model’s performance. A general ranking scheme will combine the results obtained from 10 iterations. A subset of 3 to 10 top ranking signal features will be re-evaluated and the combination of features resulting in the best classification performance will serve as the diagnostic MEG-index.

## Discussions

Evidence has accumulated that the onset of severe mental health problems in adolescence, such as psychosis, is preceded by alterations in functional and anatomical brain networks [71, 72] as well as with changes in psychological processes related to affect regulation [26]. Accordingly, further insights into the neurobiological as well as psychosocial factors that confer risk and resilience towards the development of psychosis and poor functional outcomes in CHR-individuals is an important objective.

We expect that the YouR-study will provide novel data to address these questions as well yield biomarkers that allow prediction of mental health outcomes in CHR-individuals. Specifically, we expect for the first-time a comprehensive characterization of the role of neural oscillations in emerging psychosis and its relationship to changes in E/I-balance parameters. While both rhythmic activity and alterations in GABA/Glutamatergic neurotransmission have been implicated in circuit abnormalities and cognitive deficits in established ScZ [15], their role in the early stages of psychosis is less clear. Accordingly, these data could inform novel approaches towards the at-risk mental state paradigm as well as allow the search for targeted approaches to correct abnormalities in circuit dysfunctions which could eventually lead to the prevention of severe mental health outcomes during the transition from adolescence to adulthood.

## Abbreviations

ACES: Adverse childhood experience scale
ASS: Auditory steady-state
BACS: Brief Assessment of Cognition in Schizophrenia Battery
BAPS: Beliefs About Paranoia Scale
BCSS: The Brief Core Schema Scale
BLIPs: Brief limited intermittent psychotic episodes
BS: Basic symptom
CAARMS: Comprehensive Assessment of At-Risk Mental State
CAMHS: Child and Adolescent Mental Health Services
CCNi: Centre for Cognitive Neuroimaging
CHR: Clinical high-risk criteria
CMHTs: Community Mental Health Teams
E/I: Excitatory-inhibition
ERFs: Event-related fields
ERPs: Event-related potentials
FE: First-episode
fMRI: Functional Magnetic Resonance Imaging
FROGS: Functional Remission in General Schizophrenia
GAF: Global Assessment of Functioning
GLM: Generalized Linear Model
Glx: Glutamate/Glutamine
I-PANAS-SF: International Positive and Negative Affect Schedule, short-form
MEG: Magnetocencephalography
MEGA-PRESS: MEGA point-resolved Spectroscopy
MMNm: Magnetic mismatch negativity
MNE: Minimum Norm Estimates
MNI: Montreal Neurological Institute
MRI: Magnetic Resonance Imaging
MRS: Magnetic Resonance Spectroscopy
PAM: Psychosis Attachment Measure
PCA: Principal component analysis
PCMHTs: Primary Care Mental Health Teams
PennCNP: University of Pennsylvania Computerized Neuropsychological Testing Battery
RISC: Rust Inventory of Schizotypal Cognitions
ScZ: Schizophrenia
SIPS: Structured Interview for Prodromal Symptoms
SPI-A: Schizophrenia Proneness Instrument, Adult Version
SPM: Statistical Parametric Mapping
UHR: Ultra-high risk
YouR-Study: Youth Mental Health Risk and Resilience Study
DSM: Diagnostic and Statistical Manual of the American Psychiatric Association
